# Preoperative blood urea nitrogen-to-serum albumin ratio for prediction of in-hospital mortality in patients who underwent emergency surgery for acute type A aortic dissection

**DOI:** 10.1038/s41440-024-01673-z

**Published:** 2024-05-20

**Authors:** Qingsong Wu, Jian Zheng, Jianling Lin, Linfeng Xie, Mirong Tang, Meng Ke, Liangwan Chen

**Affiliations:** 1 Department of Cardiovascular Surgery, Union Hospital, Fujian Medical University, Fuzhou, Fujian P. R. China; 2 Key Laboratory of Cardio-Thoracic Surgery (Fujian Medical University), Fujian Province University, Fuzhou, Fujian 350001 P. R. China; 3 Department of Pharmacy, the First Affiliated Hospital of Fujian Medical University, Fuzhou, Fujian P. R. China; 4 Fujian Medical University, Fuzhou, Fujian P. R. China

**Keywords:** Blood urea nitrogen-to-serum albumin ratio, Acute type A aortic dissection, Emergency surgery, Postoperative in-hospital mortality, Prognostic ability

## Abstract

The study aimed to assess the predictive value of blood urea nitrogen (BUN)-to-albumin ratio (BA-R) for in-hospital mortality in patients undergoing emergency surgery for acute type A aortic dissection (ATAAD). Patients who were diagnosed with ATAAD and underwent emergency surgery within 48 hours of onset at our hospital between January 2015 and December 2021 were included in this study. The primary endpoint of this study was postoperative in-hospital mortality (POIM). The data of the survivors and non-survivors were retrospectively compared analyses. A total of 557 ATAAD patients were included, with 505 survivors and 52 non-survivors. The preoperative BA-R of the non-survivor group was significantly higher than that of the survivor group (*P* < 0.001). Univariate regression analysis showed that preoperative BA-R, serum creatinine level, SA level, D-dimer level, age, myocardial ischemia, cerebral ischemia, and aortic clamp time were risk factors for POIM. In addition, multivariable regression analysis showed that preoperative BA-R ≥ 0.155 mmol/g was a risk factor for POIM (odds ratio, 6.815 [3.582–12.964]; *P* < 0.001). Receiver operating characteristic curve indicated that the cut-off point for preoperative BA-R was ≥0.155 mmol/g (area under the curve =0.874). The sensitivity and specificity of preoperative BA-R in predicting the POIM of patients who underwent emergency surgery for ATAAD were 84.6% and 71.3%, respectively (95% confidence interval, 0.829–0.919; *P* < 0.001). In conclusion, Preoperative BA-R is a simple, rapid, and potentially useful prognostic indicator of POIM in patients with ATAAD.

**Figure:**
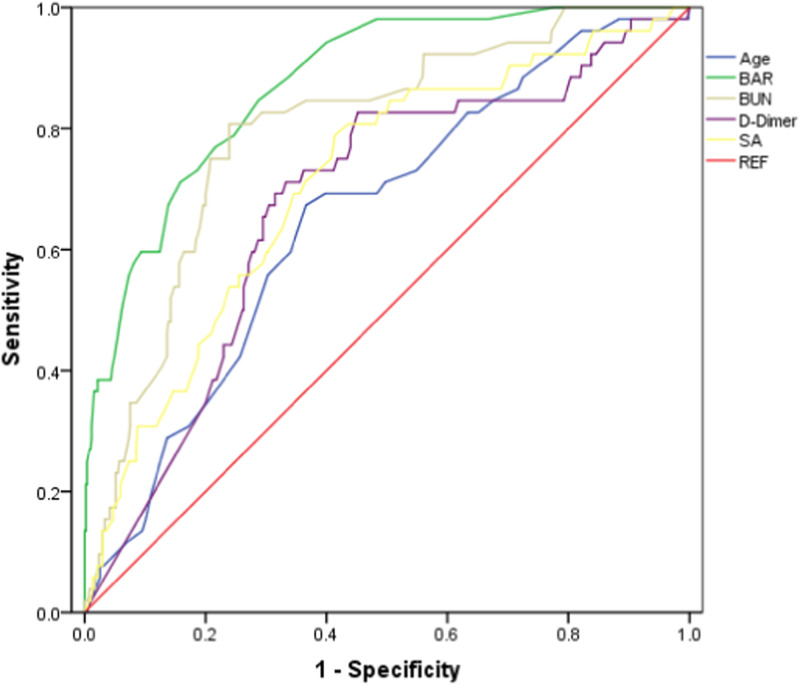
BAR: Blood urea nitrogen-to-albumin ratio, BUN: Blood urea nitrogen, SA: Serum albumin, REF: Reference. The aim of this study was to evaluate the prognostic value of BA-R for the prediction of postoperative in-hospital mortality in patients who underwent emergency surgery for ATAAD. A total of 557 patients with ATAAD were enrolled, and 505 survived while 52 did not. The preoperative BA-R of the non-survivor group was significantly higher than that of the survivor group (0.27 [0.18, 0.46] vs. 0.12 [0.10, 0.16]mmol/g; *P* < 0.001). The study showed that preoperative BA-R ≥ 0.155 mmol/g was a risk factor for POIM (odds ratio, 6.815 [3.582–12.964]; *P* < 0.001). ROC curve indicated that the cut-off point for preoperative BA-R was ≥0.155 mmol/g (AUC = 0.874) and the sensitivity and specificity were 84.6% and 71.3%, respectively (95% CI, 0.829–0.919; *P* < 0.001). We believe that our study makes a significant contribution to the literature because we found preoperative BA-R to be a simple, rapid, and potentially useful prognostic indicator of postoperative in-hospital mortality in patients with ATAAD.

BAR: Blood urea nitrogen-to-albumin ratio, BUN: Blood urea nitrogen, SA: Serum albumin, REF: Reference. The aim of this study was to evaluate the prognostic value of BA-R for the prediction of postoperative in-hospital mortality in patients who underwent emergency surgery for ATAAD. A total of 557 patients with ATAAD were enrolled, and 505 survived while 52 did not. The preoperative BA-R of the non-survivor group was significantly higher than that of the survivor group (0.27 [0.18, 0.46] vs. 0.12 [0.10, 0.16]mmol/g; *P* < 0.001). The study showed that preoperative BA-R ≥ 0.155 mmol/g was a risk factor for POIM (odds ratio, 6.815 [3.582–12.964]; *P* < 0.001). ROC curve indicated that the cut-off point for preoperative BA-R was ≥0.155 mmol/g (AUC = 0.874) and the sensitivity and specificity were 84.6% and 71.3%, respectively (95% CI, 0.829–0.919; *P* < 0.001). We believe that our study makes a significant contribution to the literature because we found preoperative BA-R to be a simple, rapid, and potentially useful prognostic indicator of postoperative in-hospital mortality in patients with ATAAD.

## Abstract (web summary)

BAR: Blood urea nitrogen-to-albumin ratio, BUN: Blood urea nitrogen, SA: Serum albumin, REF: Reference. The aim of this study was to evaluate the prognostic value of BA-R for the prediction of postoperative in-hospital mortality in patients who underwent emergency surgery for ATAAD. A total of 557 patients with ATAAD were enrolled, and 505 survived while 52 did not. The preoperative BA-R of the non-survivor group was significantly higher than that of the survivor group (0.27 [0.18, 0.46] vs. 0.12 [0.10, 0.16]mmol/g; *P* < 0.001). The study showed that preoperative BA-R ≥ 0.155 mmol/g was a risk factor for POIM (odds ratio, 6.815 [3.582–12.964]; *P* < 0.001). ROC curve indicated that the cut-off point for preoperative BA-R was ≥0.155 mmol/g (AUC = 0.874) and the sensitivity and specificity were 84.6% and 71.3%, respectively (95% CI, 0.829–0.919; *P* < 0.001). We believe that our study makes a significant contribution to the literature because we found preoperative BA-R to be a simple, rapid, and potentially useful prognostic indicator of postoperative in-hospital mortality in patients with ATAAD.

## Background

Acute type A aortic dissection (ATAAD) is a life-threatening cardiovascular disease that requires immediate management. Emergency surgery for ATAAD is challenging for cardiovascular surgeons because of its high morbidity and mortality rates [1–3]. Over the past decade, studies have shown that the in-hospital mortality rate for emergency surgery for ATAAD is higher than 20% [4, 5]. At present, some prognostic indicators and models can be used to facilitate subsequent treatment for patients with ATAAD [6, 7]. However, the complexity of ATAAD makes the prediction of its clinical outcome challenging. In addition, no sensitive, preoperative, prognostic biomarker for emergency surgery for ATAAD has been established yet.

Blood urea nitrogen (BUN) is used to reflect the relationship between kidney status, nutritional status, and protein metabolism. BUN has also been shown to be an effective biomarker of the severity and prognoses of several diseases, such as acute intracerebral hemorrhage [8], acute pancreatitis [9], and pneumonia [10]. Furthermore, BUN has been reported to be closely related to in-hospital mortality in patients with acute aortic dissection (AAD) [11]. In human serum, as a stable protein albumin is associated with inflammation, thrombosis, and platelet activation. Previous studies have shown that serum albumin (SA) level is a strong independent predictor of mortality and prognoses in cardiovascular diseases, such as AAD, heart failure and acute coronary syndrome [12–15]. However, there are no data on the relationship between BUN-to-serum albumin ratio (BA-R) and mortality in patients with ATAAD. Therefore, the aim of this study was to evaluate the prognostic value of BA-R for the prediction of postoperative in-hospital mortality (POIM) in patients who underwent emergency surgery for ATAAD.

## Methods

### Study population

This was a retrospective study of the prognostic value of BA-R for the prediction of in-hospital mortality in patients who underwent emergency surgery for ATAAD. From January 2015 to December 2021, the medical records of 557 patients who underwent emergency surgery for ATAAD at our hospital were retrospectively evaluated. The primary endpoint was POIM. Data from the survivors and non-survivors were separately compared.

**Fig. 1:**
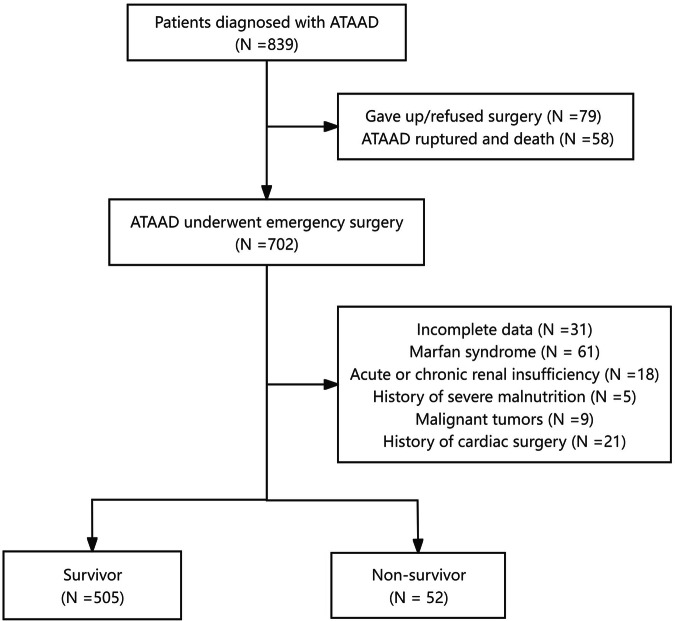
A consort type diagram of whole patients with ATAAD underwent emergency surgery

Patients with ATAAD confirmed using magnetic resonance angiography or computed tomography angiography were selected for enrolment in this study. The eligibility criteria were as follows: patients who underwent emergency tetrafurcate graft with stented elephant trunk implantation for total arch replacement surgery [16] or ascending aorta and hemiarch replacement combined with a triple-branched stent graft surgery within 48 h from the onset of ATAAD [17]. The exclusion criteria were as follows: incomplete data, Marfan syndrome, acute or chronic renal insufficiency, history of severe malnutrition or malignant tumors, or history of cardiac surgery (Fig. 1).

This study was approved by the Ethics Committee of our hospital and was conducted according to the tenets of the Declaration of Helsinki. The requirement for informed consent was waived by the Ethics Committee due to the retrospective nature of the study.

### Data collection

The clinical data of the survivor and non-survivor groups were retrospectively collated and statistically analyzed. The data collected included baseline characteristics, serum measurements, preoperative complications, surgery information, and POIM.

### Statistical analysis

Numbers or percentages for categorical variables, tested using the χ^2^ test. Median and interquartile range or mean ± standard deviation for Continuous variables and were analyzed using the T test or Mann–Whitney U test. Determined the cut-off points of continuous variables by receiver operating characteristic (ROC) curves. Categorical variables were then determined according to the cut-off points. Thereafter, univariate and multivariate regression analyses were performed. For multivariate regression analyses of related risk factors, we selected a single factor with a *P* value of <0.10. Using the SPSS 19.00 (IBM SPSS Inc., Chicago, USA). statistical software package for all data statistically analyzed. *P* values of <0.05 were considered statistically significant.

## Results

A total of 557 patients met the inclusion criteria and were included in this study. Of these, 502 (90.1%) were survivors, and 55 (9.9%) were non-survivors (Table 1).

**Table 1:** Preoperative data of the two patient groups

Variables	Group survivor (*n* = 505)	Group non-survivor (*n* = 52)	*P* value
Male, *n* (%)	377 (74.7)	38 (73.1)	0.804
Age, *n* (year)	52.0 (45.0, 61.0)	59.0 (51.0, 64.0)	<0.001
Body mass index (Kg/M^2^)	25.7 ± 4.0	26.3 ± 4.2	0.289
BUN (mmol/L)	5.20 (4.3, 7.00)	8.65 (585, 11.50)	<0.001
SA (g/L)	38.0 ± 4.0	35.8 ± 4.2	<0.001
BA-R (mmol/g)	0.14(0.11, 0.19)	0.27(0.18, 0.45)	<0.001
Serum creatinine (umol/L)	77.0 (65.0, 108.0)	94.0 (71.5, 134.3)	0.018
D-Dimer(ug/mL)	7.55 (3.46, 18.40)	17.70 (9.02, 20.0)	<0.001
Leukocyte (10ˆ9/L)	12.1 (9.3, 14.3)	12.4 (9.2, 15.7)	0.415
Platelet (10ˆ9/L)	199.1 ± 72.9	203.2 ± 70.1	0.701
Prothrombin time(sec)	13.8 (13.2, 14.6)	13.9 (13.4, 15.2)	0.537
Heamoglobin (g/L)	129.7 ± 21.1	127.4 ± 19.6	0.449
Troponin-I (ug/L)	0.6 ± 3.0	0.7 ± 2.3	0.765
NT-ProBNP (pg/mL)	278 (150.5, 591.0)	288 (163.0, 847.0)	0.523
Lactic acid (mmol/L)	1.59 ± 0.89	1.94 ± 1.35	0.012
Alanine aminotransferase (IU/L)	26.0 (18.0, 43.0)	28.0 (21.0, 53.5)	0.447
Aspartate aminotransferase (IU/L)	27.0 (19.0, 46.0)	28.0 (19.5, 67.0)	0.431
LVEF (%)	63.6 ± 6.9	62.6 ± 6.1	0.312
Hypertension, *n* (%)	388 (76.8)	42 (88.8)	0.519
Diabetes, *n* (%)	38 (7.5)	2 (3.8)	0.486
Coronary heart disease, *n* (%)	45 (89.1)	6 (11.5)	0.532
COPD, *n* (%)	15 (3.0)	2 (3.8)	0.941
Acute renal insufficiency	37 (7.3)	7 (13.5)	0.118
Myocardial ischemia	14 (2.8)	4 (7.7)	0.134
Cerebral ischemia	12 (2.4)	4 (7.7)	0.080
AR(Medium or above), *n* (%)	60 (11.9)	7 (13.5)	0.739

*BUN* blood urea nitrogen, *SA* serum albumin, *BA-R* blood urea nitrogen-to-serum albumin ratio, *LVEF* left ventricular ejection fraction, *COPD* chronic obstructive pulmonary disease, *AR* aortic regurgitation, *CPB* cardiopulmonary bypass

Continuous normally distributed variables were expressed as mean( ± standard deviation) and not-normally distributed variables as medians(interquartile range). Counts are expressed as percentages. χ^2^ test for categorical variables and wilcoxon rank sum test for continuous variables

No significant differences were noted in sex, body mass index, leukocyte count, prothrombin time, hemoglobin level, troponin-I level, NT-ProBNP level, aspartate aminotransferase level, alanine aminotransferase level, aortic regurgitation (medium or above), left ventricular ejection fraction, coronary heart disease, diabetes, hypertension, chronic obstructive pulmonary disease, acute renal insufficiency, myocardial ischemia, cerebral ischemia, operation procedure, and cardiopulmonary bypass time between the survivor and non-survivor groups. However, significant differences were observed in age (*P* < 0.001), serum creatinine level (*P* = 0.018), BUN level (*P* < 0.001), SA level (*P* < 0.001), BA-R (*P* < 0.001), D-dimer level (*P* < 0.001), lactic acid level (*P* = 0.012), operation time (*P* = 0.037), and aortic clamp time (*P* = 0.016) between the survivor and non-survivor groups. Comparisons of variables between the groups are shown in Table 1 and Table 2.

**Table 2:** Surgical data of the two patient groups

Valuables	Group survivor (*n* = 505)	Group non-survivor (*n* = 52)	*P* value
Surgical procedure			
SUN’S, *n* (%)	41 (78.9)	11 (21.1)	0.816
CHEN’S, *n* (%)	405 (80.2)	100 (19.8)	0.816
Intraoperative time			
Operative time (min)	303.5 ± 51.4	319.4 ± 59.5	0.037
CPB time (min)	140.3 ± 24.5	145.7 ± 25.6	0.130
Aortic clamp time (min)	48.9 ± 15.2	56.0 ± 19.9	0.016
Aortic Root Concomitant procedure			
Bentall, *n* (%)	92 (18.2)	8 (15.4)	0.612
Aortic valve replacement, *n* (%)	19 (3.8)	1 (1.9)	0.774
Cabrol, *n* (%)	6 (1.2)	1 (1.9)	0.841
Coronary artery bypass surgery, *n* (%)	65 (12.9)	8 (15.4)	0.609
David, *n* (%)	11 (2.2)	1 (1.9)	0.703
Aortic sinus repaired, *n* (%)	192 (38.0)	21 (40.4)	0.738

Continuous normally distributed variables were expressed as mean(± standard deviation) and not-normally distributed variables as medians(interquartile range)

SUN’S: tetrafurcate graft with stented elephant trunk implantation for total arch replacement surgery

CHEN’S: ascending aorta and hemiarch replacement combined with a triple-branched stent graft surgery. cardiopulmonary bypass (CPB)

Univariate analysis revealed that preoperative BA-R, BUN level, SA level, serum creatinine level, D-dimer level, lactic acid level, age, myocardial ischemia, cerebral ischemia, and aortic clamp time were risk factors for POIM. Further, multivariate regression analyses results showed that BA-R, BUN level, SA level, D-dimer level, and age increased the risk of POIM. The odds ratios for BA-R ≥ 0.155 mmol/g, BUN level ≥6.15 mmol/L, SA level <37.05 g/L, D-dimer ≥8.21 µg/mL, and age ≥56 years were 6.706 (2.995–15.016), 8.698 (3.641–20.776), 5.748 (2.450–13.484), 4.051 (1.640–10.008), and 3.741 (1.640–8.534), respectively (Table 3).

**Table 3:** Univariate and multivariate analyses of all patients

Variable	Univariate Model			Multivariate Model		
	OR	95% CI	*P* value	OR	95% CI	P value
Age ≥ 56 (years)	3.561	1.941–6.535	<0.001	3.741	1.640–8.534	0.002
Male	0.922	0.484–1.756	0.804	–	–	–
Body mass index ≥ 25 (kg/m^2^)	1.087	0.610–1.937	0.777	–	–	–
BUN ≥ 6.15 (mmol/L)	4.919	2.672–9.055	<0.001	8.698	3.641–20.776	<0.001
SA<37.05 (g/L)	3.294	1.688–6.426	<0.001	5.748	2.450–13.484	<0.001
BA-R ≥ 0.155 (mmol/g)	6.815	3.582–12.964	<0.001	6.706	2.995–15.016	<0.001
D-Dimer ≥ 8.21 (ug/mL)	5.805	2.771–12.160	<0.001	4.051	1.640–10.008	0.002
Prothrombin time ≥ 15.65 (sec)	1.197	0.617–2.321	0.595	–	–	–
Heamoglobin<7.04 (10ˆ9/L)	0.895	0.486–1.646	0.721	–	–	–
Hemoglobin <125.5 (g/L)	1.074	0.564–2.046	0.828	–	–	–
Platelet ≥ 222.5 (10ˆ9/L)	0.995	0.560–1.768	0.986	–	–	–
NT-ProBNP ≥ 242.0 (pg/mL)	1.159	0.618–2.175	0.646	–	–	–
Troponin-I ≥ 0.408 (ug/L)	1.247	0.537–2.899	0.608	–	–	–
Lactic acid ≥ 2.55 (mmol/L)	2.889	1.476–5.657	0.002	2.489	0.914–6.780	0.075
Alanine aminotransferase ≥ 38.5 (IU/L)	1.276	0.704–2.314	0.422	–	–	–
Aspartate aminotransferase ≥ 47.5 (IU/L)	1.170	0.629–2.176	0.619	–	–	–
Serum creatinine ≥ 96.3 (umol/L)	2.412	1.355–4.293	0.003	1.491	0.611–3.637	0.380
LVEF ≥ 62.35 (%)	0.545	0.241–1.235	0.146	–	–	–
Hypertension, presence	1.266	0.616–2.602	0.520	–	–	–
Diabetes, presence	0.492	0.115–2.099	0.338	–	–	–
Coronary heart disease, presence	0.77	0.326–1.820	0.552	–	–	–
COPD, presence	1.403	0.310–6.350	0.660	–	–	–
Myocardial ischemia, presence	3.475	1.209–9.985	0.021	1.480	0.263–8.341	0.657
Cerebral ischemia, presence	3.424	1.063–11.028	0.039	2.160	0.467–9.979	0.324
AR(Medium or above), presence	1.639	0.778–3.455	0.194	–	–	–
Surgical procedure						
SUN’S	1.067	0.436–2.613	0.887	–	–	–
CHEN’S	0.937	0.383–2.295	0.887	–	–	–
Operative time ≥ 309.2 (min)	1.590	0.897–2.818	0.112	–	–	–
CPB time ≥ 140.8 (min)	1.162	0.656–2.057	0.608	–	–	–
Aortic clamp time ≥ 49.6 (min)	1.923	1.081–3.419	0.026	1.774	0.812–3.874	0.150
Aortic Root Concomitant procedure	1.229	0.568–2.662	0.601	–	–	–

*BUN* blood urea nitrogen, *SA* serum albumin, *BA-R* blood urea nitrogen-to-serum albumin ratio, *LVEF* left ventricular ejection fraction, *COPD* chronic obstructive pulmonary disease, *AR* aortic regurgitation, *CI* confidence interval, *OR* odds ratios

SUN’S: tetrafurcate graft with stented elephant trunk implantation for total arch replacement surgery

CHEN’S: ascending aorta and hemiarch replacement combined with a triple-branched stent graft surgery. cardiopulmonary bypass (CPB)

**Fig. 2:**
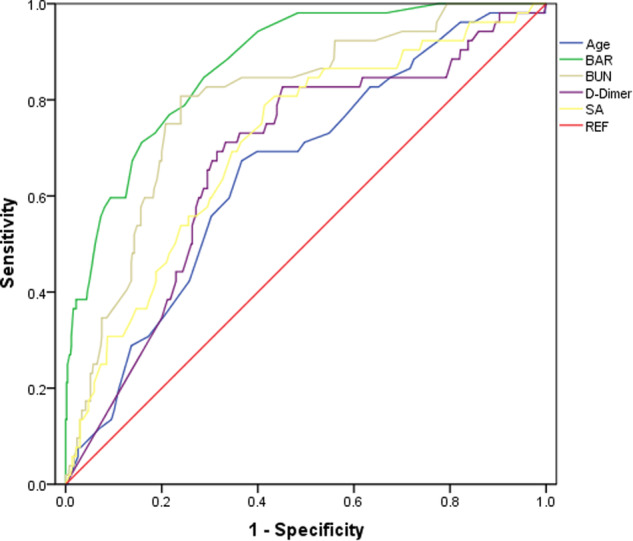
Comparison of the receiver operating characteristic curves of blood urea nitrogen level, serum albumin level, blood urea nitrogen/albumin ratio, D-dimer level, and age

The receiver operating characteristic (ROC) curve analysis showed a significant association between preoperative B-AR and POIM in patients with ATAAD (area under the curve [AUC] = 0.874, *P* < 0.001). Preoperative BA-R ≥ 0.155 mmol/g showed 84.6% sensitivity and 71.3% specificity in predicting POIM, which were the highest among all variables. Comparatively, the sensitivity and specificity of BUN level, albumin level, D-dimer level, and age were lower. Overall, the predictive performance of BA-R was significantly better than that of the other four variables (Table 4, Fig. 2).

**Table 4:** Prognostic performances of parameters for the prediction of in-hospital mortality in patients with acute type A aortic dissection

	BA-R	BUN	SA	D-dimer	Age
AUC	0.874	0.795	0.711	0.676	0.653
95% CI	0.829–0.919	0.735–0.856	0.638–0.783	0.600–0.751	0.579–0.727
p value	<0.001	<0.001	<0.001	0.002	0.002
Cut-off value	≥ 0.155 (mmol/g)	≥ 6.15 (mmol/L)	<37.05 (g/L)	≥ 8.21 (ug/mL)	≥ 56 years
Sensitivity, %	84.6	76.0	58.6	82.7	71.2
Specificity, %	71.3	80.8	78.8	54.9	63.4

*BUN* blood urea nitrogen, *SA* serum albumin, *BA-R* blood urea nitrogen-to-serum albumin ratio, *AUC* area under curve, *CI* confidence interval

## Discussion

ATAAD is a critical illness with a high POIM rate owing to the several associated postoperative complications. Many studies have investigated the postoperative prognoses of patients with ATAAD [18–22]; however, to the best of our knowledge, our study is the first to evaluate the association between BA-R and POIM in patients with ATAAD. We retrospectively analyzed the data of 557 patients who underwent emergency surgery for ATAAD at our hospital. The preoperative BA-R of the survivor group was higher than that of the non-survivor group, suggesting that. We concluded that preoperative BA-R could be an independent predictor of POIM in patients with ATAAD and that. Hence, the findings of the present study suggest that BA-R is a good predictor to identify ATAAD patients at high risk of POIM.

A previous study has shown that BUN is a biomarker that can be used for assessing blood volume insufficiency and that its concentration is associated with urea excretion, renal tubule reabsorption, and blood volume status factors [10]. The occurrence of aortic dissection disrupts the hemodynamics of the body, leading to activation of the sympathetic nervous systems and renin-angiotensin-aldosterone [23]. This results in increased angiotensin and epinephrine levels in the body, which lead to renal vasoconstriction, decreased glomerular ultrafiltration, increased water and sodium absorption, decreased urine output, and ultimately increased urea reabsorption [24]. AAD leads to reduced blood supply and hypoperfusion in the target organs involved. The release of arginine vasopressin is stimulated as a compensatory effect to maintain the balance of organ perfusion. Arginine vasopressin regulates the expression of arginine vasopressin-sensitive aquaporin by binding to its V2 receptor, which then increases the water permeability of epithelial cells, promotes water reabsorption, and further increases urea reabsorption [25]. Previous studies have shown that elevated BUN level is associated with mortality in various diseases and conditions [9–11, 26–30]. However, BUN is synthesized in the liver through protein catabolism and is affected by protein intake and consumption; thus, it is affected not only by neurohumoral activity but by albumin level as well.

Albumin level may be related to liver synthesis, body catabolism, and vascular function [31]. Various physiological properties of albumin have been reported, including maintenance of plasma colloid osmolality, anti-inflammatory and antioxidant effects, and initiation of platelet activation [32–35]. In physiological homeostasis SA plays the leading role by participating in anti-inflammatory functions, alleviating ischemia-reperfusion injury, improving arterial hyporeactivity and maintaining normal colloid osmotic pressure [35–37]. Inflammation plays an important role in the development and prognosis of AAD. In addition, the severity of inflammation has been shown to be related to aortic dissection rupture. Patients with type B AAD who do not survive have lower SA levels than survivors. Low SA level has also been reportedly associated with early adverse complications and long-term mortality [13, 38]. Keskin et al. focused on the prognostic nutritional index as a predictor of in-hospital mortality in patients with ATAAD and reported that lower prognostic nutritional index values, which incorporate albumin levels, were independently associated with higher in-hospital mortality rates. Further, they reported significantly lower SA levels in the non-survivor group than in the survivor group, concluding that lower prognostic nutritional index values are independently associated with in-hospital mortality in ATAAD [39]. We deduced that this may have been associated with decreased SA synthesis and increased catabolism associated with inflammation [40]. This is consistent with the results of the present study, which indicate that non-survivors have significantly lower albumin levels than survivors. SA is an important inhibitor of platelet activation and aggregation; therefore, decreased albumin levels in pathological conditions lead to increased platelet activation and aggregation, resulting in an increased risk of associated thrombotic events [31]. A previous study has reported that platelets play an important role in thrombosis, and that thrombosis can reflect the early prognosis of AAD [41]. Further, studies have also shown that platelet count, ratio of mean platelet volume to platelet count, and platelet activity markers are the independent predictors of mortality in patients with AAD, and that low SA levels, thrombosis, and platelet activation are associated with an increased risk of death in AAD [42, 43]. However, the mechanisms underlying the association between reduced SA level and mortality in patients with ATAAD remain unclear.

Although the results of this study suggest that elevated D-dimer level and older age are also predictors of a high risk of POIM in patients with ATAAD, their predictive powers were inferior to that of BA-R. The use of BA-R as a prognostic biomarker in the present study allowed the team of cardiovascular surgeons to evaluate the clinical statuses of the patients with ATAAD from two different perspectives. BUN and SA complement each other, influence each other, and are closely related. In addition, measurement of these indicators in clinical practice is simple, rapid, and economical. Thus, BA-R has considerable predictive value for POIM in patients undergoing emergency surgery for ATAAD.

### Limitations

This study has some limitations. First, we only measured the BUN and SA levels before emergency surgery. The postoperative changes in BUN and SA levels were not assessed. Since BUN and SA levels may change over time, assessment of these changes may yield more accurate results than single measurements. Thus, BUN and SA levels should be monitored dynamically. Second, we did not obtain information on inflammatory indicators, such as C-reactive protein, serum interleukin-6, and procalcitonin, or nutritional indicators, such as prealbumin, which might be helpful for exploring the mechanism underlying the association between preoperative BA-R and the prognoses of patients with ATAAD. Finally, this was a single-center, retrospective analysis with a limited sample size that might limit statistical power. Nevertheless, this study produced some significant and valuable results.

## Conclusion

Preoperative BA-R is a simple, rapid, and potentially useful prognostic factor of POIM in patients with ATAAD.

## Availability of data and materials

All data generated or analysed during this study are included in this published article.

## References

[1] Nienaber CA, Clough RE (2015). Management of acute aortic syndrome. Lancet.

[2] Erbel R, Aboyans V, Boileau C, Bossone E, Di Bartolomeo R, Eggebrecht H (2014). 2014 ESC Guidelines on the diagnosis and treatment of aortic diseases. Kardiol Pol.

[3] Evangelista A, Isselbacher EM, Bossone E, Gleason TG, Eusanio MD, Sechtem U (2018). IRAD Investigators. Insights from the international registry of acute aortic dissection: a 20-year experience of collaborative clinical research. Circulation.

[4] Pape LA, Awais M, Woznicki EM, Suzuki T, Trimarchi S, Evangelista A (2015). Presentation, diagnosis, and outcomes of acute aortic dissection: 17-year trends from the international registry of acute aortic dissection. J Am Coll Cardiol.

[5] Zhu Y, Lingala B, Baiocchi M, Tao JJ, Toro Arana V, Khoo JW (2020). Type A aortic dissection-experience over 5 decades: JACC historical breakthroughs in perspective. J Am Coll Cardiol.

[6] Ghoreishi M, Wise ES, Croal-Aprahams L, Tran D, Pasrija C, Drucker CB (2018). A novel risk score predicts operative mortality after acute type A aortic dissection repair. Ann Thorac Surg.

[7] Wu Q, Li J, Chen L, Yan LL, Qiu Z, Shen Y (2020). Efficacy of interleukin-6 in combination with D-dimer in predicting early poor postoperative prognosis after acute stanford type a aortic dissection. J Cardiothorac Surg.

[8] Rhoney DH, Parker D, Millis SR, Whittaker P (2012). Kidney dysfunction at the time of intracerebral hemorrhage is associated with increased in-hospital mortality: a retrospective observational cohort study. Neurol Res.

[9] Koutroumpakis E, Wu BU, Bakker OJ, Dudekula A, Singh VK, Besselink MG (2015). Admission hematocrit and rise in blood urea nitrogen at 24h outperform other laboratory markers in predicting persistent organ failure and pancreatic necrosis in acute pancreatitis: a post Hoc analysis of three large prospective databases. Am J Gastroenterol.

[10] Ryu S, Oh SK, Cho SU, You Y, Park JS, Min JH (2021). Utility of the blood urea nitrogen to serum albumin ratio as a prognostic factor of mortality in aspiration pneumonia patients. Am J Emerg Med.

[11] Liu J, Sun LL, Wang J, Ji G (2018). Blood urea nitrogen in the prediction of in-hospital mortality of patients with acute aortic dissection. Cardiol J.

[12] Gao Y, Li D, Cao Y, Zhu X, Zeng Z, Tang L (2019). Prognostic value of serum albumin for patients with acute aortic dissection: A retrospective cohort study. Med (Baltim).

[13] Zeng R, Li D, Deng L, He Y, Sun X, Wan Z (2016). Hypoalbuminemia predicts clinical outcome in patients with type B acute aortic dissection after endovascular therapy. Am J Emerg Med.

[14] Oduncu V, Erkol A, Karabay CY, Kurt M, Akgün T, Bulut M (2013). The prognostic value of serum albumin levels on admission in patients with acute ST-segment elevation myocardial infarction undergoing a primary percutaneous coronary intervention. Coron Artery Dis.

[15] Liu M, Chan CP, Yan BP, Zhang Q, Lam YY, Li RJ (2012). Albumin levels predict survival in patients with heart failure and preserved ejection fraction. Eur J Heart Fail.

[16] Ma WG, Zhu JM, Zheng J, Liu YM, Ziganshin BA, Elefteriades JA (2013). Sun’s procedure for complex aortic arch repair: total arch replacement using a tetrafurcate graft with stented elephant trunk implantation. Ann Cardiothorac Surg.

[17] Chen LW, Wu XJ, Dai XF, Liao DS, Li C, Wang QM (2015). A self-adaptive triple-branched stent graft for arch repair during open type A dissection surgery. J Thorac Cardiovasc Surg.

[18] Gomibuchi T, Seto T, Komatsu M, Tanaka H, Ichimura H, Yamamoto T (2018). Impact of frailty on outcomes in acute type A aortic dissection. Ann Thorac Surg.

[19] Orrico M, Ronchey S, Praquin B, Setacci C, Lachat M, Mangialardi N (2019). VI2TA2 S2C2ORE: a new score system for in hospital mortality in acute aortic dissections. J Cardiovasc Surg (Torino).

[20] Lin Y, Chen Q, Peng Y, Chen Y, Huang X, Lin L (2021). Prognostic nutritional index predicts in-hospital mortality in patients with acute type A aortic dissection. Heart Lung.

[21] Yamasaki M, Yoshino H, Kunihara T, Akutsu K, Shimokawa T, Ogino H (2021). Risk analysis for early mortality in emergency acute type A aortic dissection surgery: experience of Tokyo Acute Aortic Super-network. Eur J Cardio-Thorac Surg.

[22] Luehr M, Merkle-Storms J, Gerfer S, Li Y, Krasivskyi I, Vehrenberg J (2021). Evaluation of the GERAADA score for prediction of 30-day mortality in patients with acute type A aortic dissection. Eur J Cardio-thorac Surg.

[23] Zhipeng HU, Zhiwei W, Lilei YU, Hao Z, Hongbing W, Zongli R (2014). Sympathetic hyperactivity and aortic sympathetic nerve sprouting in patients with thoracic aortic dissection. Ann Vasc Surg.

[24] Li Y, Hu J, Qian H, Gu J, Meng W, Zhang EY (2015). Novel findings: Expression of angiotensin-converting enzyme and angiotensin-converting enzyme 2 in thoracic aortic dissection and aneurysm. J Renin Angiotensin Aldosterone Syst.

[25] Schrier RW (2008). Vasopressin and aquaporin 2 in clinical disorders of water homeostasis. Semin Nephrol.

[26] Aronson D, Mittleman MA, Burger AJ (2004). Elevated blood urea nitrogen level as a predictor of mortality in patients admitted for decompensated heart failure. Am J Med.

[27] Richter B, Sulzgruber P, Koller L, Steininger M, El-Hamid F, Rothgerber DJ (2019). Blood urea nitrogen has additive value beyond estimated glomerular filtration rate for prediction of long-term mortality in patients with acute myocardial infarction. Eur J Intern Med.

[28] Tatlisu MA, Kaya A, Keskin M, Avsar S, Bozbay M, Tatlisu K (2017). The association of blood urea nitrogen levels with mortality in acute pulmonary embolism. J Crit Care.

[29] Shorr AF, Sun X, Johannes RS, Yaitanes A, Tabak YP (2011). Validation of a novel risk score for severity of illness in acute exacerbations of COPD. Chest.

[30] Emami A, Javanmardi F, Rajaee M, Pirbonyeh N, Keshavarzi A, Fotouhi M (2019). Predictive biomarkers for acute kidney injury in burn patients. J Burn Care Res.

[31] Li HH, Xu J, Wasserloos KJ, Li J, Tyurina YY, Kagan VE (2011). Cytoprotective effects of albumin, nitrosated or reduced, in cultured rat pulmonary vascular cells. Am J Physiol Lung Cell Mol Physiol.

[32] Don BR, Kaysen G (2004). Serum albumin: relationship to inflammation and nutrition. Semin Dial.

[33] Roche M, Rondeau P, Singh NR, Tarnus E, Bourdon E (2008). The antioxidant properties of serum albumin. FEBS Lett.

[34] Lam FW, Cruz MA, Leung HC, Parikh KS, Smith CW, Rumbaut RE (2013). Histone induced platelet aggregation is inhibited by normal albumin. Thromb Res.

[35] Nicholson JP, Wolmarans MR, Park GR (2000). The role of albumin in critical illness. Br J Anaesth.

[36] Meziani F, Kremer H, Tesse A, Baron-Menguy C, Mathien C, Mostefai HA (2007). Human serum albumin improves arterial dysfunction during early resuscitation in mouse endotoxic model via reduced oxidative and nitrosative stresses. Am J Pathol.

[37] Yao X, Miao W, Li M, Wang M, Ma J, Wang Y (2010). Protective effect of albumin on VEGF and brain edema in acute ischemia in rats. Neurosci Lett.

[38] Cifani N, Proietta M, Tritapepe L, Di Gioia C, Ferri L, Taurino M (2015). Stanford-A acute aortic dissection, inflammation, and metalloproteinases: a review. Ann Med.

[39] Keskin HA, Kurtul A, Esenboğa K, Çiçek MC, Katırcıoğlu SF (2021). Prognostic nutritional index predicts in-hospital mortality in patients with acute Stanford type A aortic dissection. Perfusion.

[40] Artigas A, Wernerman J, Arroyo V, Vincent JL, Levy M (2016). Role of albumin in diseases associated with severe systemic inflammation: pathophysiologic and clinical evidence in sepsis and in decompensated cirrhosis. J Crit Care.

[41] Li DZ, Yu J, Du RS, Zeng R, Zeng Z (2016). Thrombo-inflammatory status and prognosis of acute type A aortic dissection. Herz.

[42] Li DZ, Chen QJ, Sun HP, Zeng R, Zeng Z, Gao XM (2016). Mean platelet volume to platelet count ratio predicts in-hospital complications and long-term mortality in type A acute aortic dissection. Blood Coagul Fibrinolysis.

[43] Huang B, Tian L, Fan X, Zhu J, Liang Y, Yang Y (2014). Low admission platelet counts predicts increased risk of in-hospital mortality in patients with type A acute aortic dissection. Int J Cardiol.

